# Big data - a 21st century science Maginot Line? No-boundary thinking: shifting from the big data paradigm

**DOI:** 10.1186/s13040-015-0037-5

**Published:** 2015-02-06

**Authors:** Xiuzhen Huang, Steven F Jennings, Barry Bruce, Alison Buchan, Liming Cai, Pengyin Chen, Carole L Cramer, Weihua Guan, Uwe KK Hilgert, Hongmei Jiang, Zenglu Li, Gail McClure, Donald F McMullen, Bindu Nanduri, Andy Perkins, Bhanu Rekepalli, Saeed Salem, Jennifer Specker, Karl Walker, Donald Wunsch, Donghai Xiong, Shuzhong Zhang, Yu Zhang, Zhongming Zhao, Jason H Moore

**Affiliations:** 1 Department of Computer Science, Arkansas State University, Jonesboro, AR 72467 USA; 2 Sector3 Informatics, Marana, AZ 85658 USA; 3 Sustainable Energy & Education Research Center, University of Tennessee at Knoxville, Knoxville, TN 37996 USA; 4 Department of Microbiology, University of Tennessee, Knoxville, TN 37996 USA; 5 Department of Computer Science, University of Georgia, Athens, GA 30602 USA; 6 Crop, Soil, and Environmental Sciences, University of Arkansas at Fayetteville, Fayetteville, AR 72701 USA; 7 Arkansas Biosciences Institute, Department of Biological Sciences, Arkansas State University, Jonesboro, AR 72467 USA; 8 Division of Biostatistics, School of Public Health, University of Minnesota, Minneapolis, MN 55455 USA; 9 BIO5 Institute & iPlant Collaborative, The University of Arizona, PO Box 210240, Tucson, AZ 85721 USA; 10 Department of Statistics, Northwestern University, Evanston, IL 60208 USA; 11 Center for Applied Genetic Technologies, The University of Georgia, Athens, GA 30602 USA; 12 Arkansas Science & Technology Authority, Arkansas NSF EPSCoR, Little Rock, AR 72201 USA; 13 Arkansas High Performance Computing Center, University of Arkansas at Fayetteville, Fayetteville, AR 72701 USA; 14 Department of Basic Sciences, College of Veterinary Medicine, Mississippi State University, Jackson, MS 39762 USA; 15 Department of Computer Science and Engineering, Mississippi State University, Jackson, MS 39762 USA; 16 National Institute for Computational Sciences, Department of Electrical Engineering and Computer Science, UTK and ORNL, Oak Ridge, TN 37832 USA; 17 Department of Computer Science, North Dakota State University, Fargo, ND 58102 USA; 18 Graduate School of Oceanography, University of Rhode Island, Narragansett, RI 02882 USA; 19 Department of Computer Science, University of Arkansas at Pine Bluff, Arkansas, 71601 USA; 20 Department of Electrical & Computer Engineering, Missouri University of Science & Technology, Rolla, MO 65409 USA; 21 Department of Pharmacology and Toxicology, Medical College of Wisconsin, Milwaukee, WI 53223 USA; 22 Department of Industrial and Systems Engineering, University of Minnesota, Minneapolis, MN 55455 USA; 23 Department of Computer Science, Trinity University, San Antonio, TX 78212 USA; 24 Department of Biomedical Informatics, Vanderbilt University School of Medicine, Nashville, TN 37203 USA; 25 Department of Genetics, Geisel School of Medicine, Dartmouth College, Lebanon, NH 03756 USA

**Keywords:** Big data, Maginot Line, No-Boundary thinking

## Abstract

Whether your interests lie in scientific arenas, the corporate world, or in government, you have certainly heard the praises of big data: Big data will give you new insights, allow you to become more efficient, and/or will solve your problems. While big data has had some outstanding successes, many are now beginning to see that it is not the Silver Bullet that it has been touted to be. Here our main concern is the overall impact of big data; the current manifestation of big data is constructing a Maginot Line in science in the 21st century. Big data is not “lots of data” as a phenomena anymore; The big data paradigm is putting the spirit of the Maginot Line into lots of data. Big data overall is disconnecting researchers and science challenges. We propose No-Boundary Thinking (NBT), applying no-boundary thinking in problem defining to address science challenges.

## The myth of big data

Big data has been largely promoted as a paradigm [1], bringing new challenges and opportunities. There are many national and international initiatives and funding programs [2,3] which focus on big data. The NIH definition of bioinformatics is essentially based on data: “Research, development, or application of computational tools and approaches for expanding the use of biological, medical, behavioral or health data, including those to acquire, store, organize, archive, analyze, or visualize such data [4]”. On the back cover of *The Fourth Paradigm*: *Data*-*Intensive Scientific Discovery* [1], Microsoft Corporation’s founder, Bill Gates, states “The impact of Jim Gray’s thinking is continuing to get people to think in a new way about how data and software are redefining what it means to do science.” There are many research projects and publications focused on big data; there are many big data-centered conferences and workshops; there are many big data hardware and software companies [5]; and there are many big data, high-throughput technologies, such as sequencing and imaging technologies. Big data seems to be getting more and more attention.

However, is the big data paradigm building a scientific Maginot Line in the 21^st^ century?

*The Maginot Line*

*The French*, *based on the experiences of a previous generation during World War I*, *built a line of rigid fortifications along their border with Germany just prior to the start of World War II. While hailed as a work of genius at the time*, *by the time it was built*, *offensive military tactics turned it obsolete as the German*’*s Blitzkrieg simply bypassed those fortifications. By giving the French a false sense of security*, *the Maginot Line had the effect of draining resources from more flexible defensive strategies. The return on investment turned out to be poor. Is the big data paradigm turning out to be a science Maginot Line*? *Only time will tell*, *but we are already becoming aware of the limitations of this strategy*.

Big data, admittedly, is a phenomenon. Our main concern with big data is its overall impact to our current and next generation of students and researchers: Pushing big data as a paradigm, promoting big data as a necessity in life sciences, and calling for analytical approaches to big data, these are problematic. Big data is driving a wedge between scientists of different disciplines, especially computational scientists and life scientists by focusing on the data, not the problem to be solved. A false belief in the standalone power of data separates computational scientists from the underlying problem and provides “answers” to life scientists that may be devoid of meaning. Big data is attracting the attention of our researchers and our students away from real scientific challenges. Big data, e.g., The Cancer Genome Atlas (TCGA), may have produced some good results published in Nature or Science [6], but big data overall is disconnecting researchers and science challenges.

*The Cancer Genome Atlas* (*TCGA*)

*TCGA*, *with the goal to cover more than 20 different types of human cancers* (>*11*,*000 cases*), *is collecting data from different high*-*throughput platforms* (*including gene expression profiling*, *copy*-*number variation profiling*, *SNP genotyping*, *genome*-*wide DNA methylation profiling*, *microRNA profiling*, *and exon sequencing*) *and then releasing data usually after their analysis and publications. For the pilot project and phase II of TCGA*, *about* US$200-million *has been invested in this effort to gather samples*, *generate data*, *and analyze the data. TCGA publications*, *almost all in top*-*tier science journals and almost all with the similar titles as* “*Comprehensive Molecular Characterization of X Cancer*” *or* “*Comprehensive Molecular Profiling of Y Cancers*,” *for the most part present* “*stories*” *of their data generation and data analysis with some* “*plausible*” *results. If TCGA*, *with a comprehensive team of scientists and technology experts*, *could not dig the* “*gold*” *out of the collected large amount of data*, *how could other researchers be expected to do so*? *Furthermore*, *while collected data is static*, *the human genome is dynamic. So*, *should we continue collecting more and more data with the hope of digging out the* “*gold*” *information to save patients*? *Or*, *should we think about redirecting our efforts to specific*, *science*-*driven approaches*, *dynamic and systematic*, *to save dying patients from whom we collect the data*? *What is needed are not more reports*, *more lists of publications*, *more software packages*, *and more data. Efforts like TCGA are reaching the* “*bottleneck*;” *it is hard to make significant breakthroughs in scientific challenges by focusing on big data. Since interdisciplinary research does not work well*, *how about post*-*interdisciplinary approaches such as transdisciplinary approaches* [*communication with a senior scientist*]? *Since many current methods and approaches are generic*, *how about looking into more granular layers and finely*-*detailed approaches*?

Many authors are beginning to point out the limitations of big data and that big data is not effective in solving certain problems (see the following several references [7-9]). “Big data has arrived, but big insights have not” [9].

[7] *NY Times*: [*Eight* (*No*, *Nine*!) *Problems With Big Data*]: “*Big data is here to stay*, *as it should be. But let*’*s be realistic*: *It*’*s an important resource for anyone analyzing data*, *not a silver bullet*”.

[8] *FT article*: [*Big data*: *are we making a big mistake*?]: “*Big datahas arrived*, *but big insights have not. The challenge now is to solve new problems and gain new answers* – *without making the same old statistical mistakes on a grander scale than ever*”.

[9] *RD article*: [*Why Big Data Isn*'*t the Big Problem for Genomic Medicine*]: “*Of course*, *as this technology is adopted more broadly it will deliver new challenges in data management and analytics. But it*’*s nothing this industry can*’*t handle. The true barrier to clinical adoption of genomic medicine isn*’*t data volume or scale*, *but how to empower physicians from a logistical and clinical genomics knowledge standpoint*, *while proving the fundamental efficacy of genomics medicine in terms of improved patient diagnosis*, *treatment regimens*, *outcomes and improved patient management*”.

Our main concern is not the ineffectiveness of big data for specific scientific problems. Also, our main concern is not for the numerous projects where big data seems to introduce significant false-positive results and potentially misleading discoveries (e.g., Cancer and chemotherapy are associated with a reduced Alzheimer’s risk [10]). Of course, specific projects may really need to collect big data to achieve the goals and to enable discoveries; our main concern here is not evaluating the need for big data in individual projects.

Overall, we are concerned that the big data paradigm has taken a whole generation of science and research down the wrong path and given a false sense of progress, in effect, creating a modern-day Maginot Line.

*The Maginot Line gave France a false sense of security* (*since it seems strong and big*); *Is big data giving us a false sense of security*, *by assuming we could answer science challenges by looking at big data*? *The Maginot Line gave France a wrong impression of challenge* (*see how the German army attacked it*); *Is big data a real challenge*? *Big data may not be the challenge. It is the time we should re*-*focus on the science challenge*, *which is the real challenge*.

## No-boundary thinking

We are proposing No-Boundary Thinking (NBT) to address real scientific challenges and to help science advance. Last year, we introduced the NBT concept [11]. Rather than looking for big data or software tools to provide a connection among researchers of related disciplines, with NBT, the connection will come about by defining scientific problems to address science challenges. There are many problems based on big data approaches: not only is it just ineffective, but also it is disconnecting researchers from understanding the real science challenges. Currently the core of NBT is applying no-boundary thinking in problem defining.

NBT is not just adjusting the starting point from problem solving to problem defining, either. And it is not just starting earlier with interdisciplinary research. NBT is integrating life sciences and the computational and mathematical sciences closely and inseparably through no-boundary thinking. All researchers who bring similar and complementary interests and skills need to be integrated into problem defining as well as solving. NBT is also different from “multidisciplinary” or “transdisciplinary”; it is conceptualized without disciplinary limitations or boundaries (i.e., “discipline-free”). An article that explains these concepts and provides a detailed description of NBT uniqueness is in preparation.

Several decades ago with the boost of computers and software, there might have been a point to advocate for data and software for empowering science or to promote big data and software tools to connect researchers of different disciplines. However, today in the 21st century, the overall impact of the focus on big data and software is misleading and confusing to researchers and students, making their strategies rigid, which later on will have even broader negative impacts to science in science history.
